# Functional connectivity between interoceptive brain regions is associated with distinct health‐related domains: A population‐based neuroimaging study

**DOI:** 10.1002/hbm.26275

**Published:** 2023-03-20

**Authors:** Alexander Luettich, Carolin Sievers, Fidel Alfaro Almagro, Micah Allen, Saad Jbabdi, Stephen M. Smith, Kyle T. S. Pattinson

**Affiliations:** ^1^ Nuffield Department of Clinical Neurosciences University of Oxford Oxford UK; ^2^ Wellcome Centre for Integrative Neuroimaging University of Oxford Oxford UK; ^3^ Center of Functionally Integrative Neuroscience Aarhus University Aarhus Denmark; ^4^ Aarhus Institute of Advanced Studies Aarhus University Aarhus Denmark; ^5^ Cambridge Psychiatry University of Cambridge Cambridge UK

**Keywords:** arousal and affect and cardiovascular health, body mass, functional connectivity, interoception, respiratory health, subjective health

## Abstract

Interoception is the sensation, perception, and integration of signals from within the body. It has been associated with a broad range of physiological and psychological processes. Further, interoceptive variables are related to specific regions and networks in the human brain. However, it is not clear whether or how these networks relate empirically to different domains of physiological and psychological health at the population level. We analysed a data set of 19,020 individuals (10,055 females, 8965 males; mean age: 63 years, age range: 45–81 years), who have participated in the UK Biobank Study, a very large‐scale prospective epidemiological health study. Using canonical correlation analysis (CCA), allowing for the examination of associations between two sets of variables, we related the functional connectome of brain regions implicated in interoception to a selection of nonimaging health and lifestyle related phenotypes, exploring their relationship within modes of population co‐variation. In one integrated and data driven analysis, we obtained four statistically significant modes. Modes could be categorised into domains of arousal and affect and cardiovascular health, respiratory health, body mass, and subjective health (all *p* < .0001) and were meaningfully associated with distinct neural circuits. Circuits represent specific neural “fingerprints” of functional domains and set the scope for future studies on the neurobiology of interoceptive involvement in different lifestyle and health‐related phenotypes. Therefore, our research contributes to the conceptualisation of interoception and may lead to a better understanding of co‐morbid conditions in the light of shared interoceptive structures.

## Graphical abstract

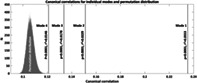

## 1 INTRODUCTION

Interoception is defined as the sensing, interpretation and integration of internal states for the maintenance of homeostasis (Azzalini et al., 2019; Chen et al., 2021; Petzschner et al., 2021; Tsakiris & Critchley, 2016). Such a process is necessarily flexible and dynamic and needs to be understood neurally in combination with body regulation through the integrated processing of the peripheral and central nervous system (Benarroch, 1993; Saper, 2002) in the context of continuous reciprocal influences of afferent and efferent pathways across unconscious and conscious levels, linking the body and the brain (Berntson & Khalsa, 2021). Mechanistically, the central nervous system may form a model of the body within the world based on both interoceptive and exteroceptive experiences and motivational states (Allen, 2020; Barrett & Simmons, 2015; Seth et al., 2012). The proposed mechanism continuously predicts and regulates internal physiological and mental states and is at the same time updated according to incoming sensations (Petzschner et al., 2021).

Regulation of cardiac, digestive and respiratory function are closely related to interoceptive processes (Chen et al., 2021), as are emotional (Craig, 2002; Critchley & Garfinkel, 2017; Damasio et al., 2000; James, 1994; Lange & Haupt, 1922; Seth & Friston, 2016), cognitive (Critchley & Garfinkel, 2018; Dunn et al., 2010; Piech et al., 2010; Sütterlin et al., 2013; Umeda et al., 2016) and reflexive, self‐related states (Craig, 2009; Quigley et al., 2021; Seth & Tsakiris, 2018).

Interoceptive ability is potentially a key mediator of health‐relevant variables, such as individual differences in physiological functioning and symptom perception. Disturbed interoception is implicated in somatic, developmental, neurological, neurodegenerative, and notably also psychiatric conditions including for instance disorders of self‐awareness, anxiety, and depression (Bonaz et al., 2021; Khalsa et al., 2018; Owens et al., 2018).

Currently, it is far from clear whether or how specific neural networks relevant to interoception relate empirically to different physiological, psychological and generally health‐related domains, that is, whether they show sensitive individual ‘fingerprints’, at the population level. To date no large‐scale studies have been done on this. Thus, we utilised a large‐scale database, the UK Biobank Imaging Study (Sudlow et al., 2015), selected brain regions which have in previous research been associated with interoception and related the resting functional connectome of these regions to a selection of lifestyle and health‐related phenotypes in order to explore their relationship within modes of population co‐variation (Smith et al., 2015) and guide future experimental interoception research. By not employing a specific task, we could relate functional connectivity to different health‐related fields in one integrative analysis, delineating shared and separated mechanisms.

## 2 METHODS AND MATERIALS

### 2.1 Participants

UK Biobank has received research ethics approval from the North West Multi‐centre Research Ethics Committee (MREC). More detailed information about UKB participants can be accessed elsewhere (Littlejohns et al., 2020; Sudlow et al., 2015). In brief, UKB contains a cohort of 500 k participants recruited from 2006 to 2010, who were registered with the UK's National Health Service (NHS) and have been sent postal invitations. Extensive sociodemographic, health‐related, and lifestyle data were collected from these participants across 22 centres in the United Kingdom. From 2014, participants were invited to take part in multimodal imaging including brain imaging at one of three dedicated UK imaging centres. Multimodal imaging is ongoing. Eligibility criteria broadly involve issues of magnet safety and tolerability (see Littlejohns et al., 2020 for more details). We accessed relevant imaging and nonimaging variables from available subjects in April 2018, and newly available imaging data in September 2018. Only complete imaging data sets were used, resulting in the exclusion of two imaging data sets. Imaging and nonimaging data were then matched and available in a total of 19,020 participants (10,055 females, 8965 males age: mean 63 years, standard deviation ±7 years, range 45–81 years).

### 2.2 Imaging

#### 2.2.1 Acquisition

Low sample size is a common problem in neuroscience research, impacting reproducibility and scientific progress (Button et al., 2013). UK Biobank is a population level prospective epidemiological health study and in addition the world's largest multimodal imaging study, including brain imaging data (Littlejohns et al., 2020). In the present study, we used resting‐state functional magnetic resonance imaging (rfMRI) data from UK Biobank to assess resting‐state functional connectivity within a connectome relevant to interoception. UK Biobank's imaging protocols can be accessed online (http://biobank.ctsu.ox.ac.uk/crystal/refer.cgi?id=2367). Further information has been published elsewhere regarding brain imaging in UK Biobank (Miller et al., 2016) and the processing pipeline (Alfaro‐Almagro et al., 2018). rfMRI data were acquired at three identical Biobank imaging centres across the United Kingdom, using a 3 T Siemens Skyra scanner with a 32‐channel head coil. The T2*‐weighted images with BOLD contrast were measured with gradient‐echo echo‐planar imaging (GE‐EPI) with ×8 multi‐slice (multiband) acceleration, no in‐plane acceleration, flip angle = 52°, fat saturation, TR = 735 ms, TE = 39 ms, FOV = 88 × 88 × 64, voxel size = 2.4 mm (isotropic). Resting‐state scans lasted 6 min, comprising 490 time points.

#### 2.2.2 rfMRI processing

We analysed data that had undergone standard pre‐processing, as follows: As described in the imaging documentation, rfMRI data were pre‐processed using FSL (the FMRIB Software Library, https://fsl.fmrib.ox.ac.uk/fsl/fslwiki). Motion correction MCFLIRT (Jenkinson et al., 2002), grand‐mean intensity normalisation, high‐pass temporal filtering (Gaussian‐weighted least‐squares straight line fitting, sigma = 50s), EPI unwarping and gradient distortion correction (GDC) were applied. EPI and GDC unwarping both included alignment to the T1 structural image using FLIRT (Jenkinson et al., 2002; Jenkinson & Smith, 2001). Images were nonlinearly warped to MNI152 standard space utilising FNIRT (Andersson et al., 2007a; Andersson et al., 2007b). Finally, artefacts were removed using independent‐component analysis (ICA) (Beckmann & Smith, 2004) followed by FMRIB's ICA‐based X‐noiseifier (FIX). All structured artefactual ICA components identified by FIX were removed, which may, for example, include components related to movement, white matter fluctuations, susceptibility, cardiac pulsation or arterial contribution, large veins, or MRI acquisition issues (Griffanti et al., 2014; Salimi‐Khorshidi et al., 2014). FIX‐cleaned time series were used to compute the functional connectivity matrix within our network of interest.

#### 2.2.3 Imaging‐derived phenotypes (IDPs)

We created binary region of interest masks (see Table 1 for additional information) for 11 regions representing the nodes of a network relevant to interoception. Insular cortex (IC) has a multisensory, integrative role, from lower‐level interoception to more higher‐level interoceptive states including feeling states and awareness, where posterior (pIC) and anterior (aIC) sections can functionally and structurally be differentiated (Allen, 2020; Craig, 2009; Critchley et al., 2004; Kurth et al., 2010; Wiech et al., 2014). The latter is often co‐activated with anterior cingulate cortex (ACC) (Craig, 2009; Menon, 2015), which can functionally be subdivided into a ventral portion (vACC) associated with internal states including autonomic function, emotion, and the self (Hamani et al., 2011; Joyce & Barbas, 2018; Qin & Northoff, 2011), and a dorsal portion (dACC). Interoceptive awareness is linked to dACC, dorsomedial prefrontal cortex (dmPFC), IC, and sensorimotor structures (Critchley et al., 2004; Khalsa et al., 2009; Pollatos et al., 2007), where we selected primary sensory (S1) and motor (M1) cortex. Ventromedial prefrontal cortex (vmPFC) situated next to vACC plays a major role in integrating interoception, affect, self‐related processing, and physiology (Koban et al., 2021; Roy et al., 2012). We also included subcortical amygdala (Amy), which receives viscerosensory input, is associated with visceromotor function and emotional processing (Bohus et al., 1996; McDougall et al., 2017; Öhman, 2005; Phelps & LeDoux, 2005) and shows extensive reciprocal connections with IC (Mufson et al., 1981), and the periaqueductal grey (PAG). The PAG is a midbrain structure that has been linked to interoception and autonomic function, where lateral (lPAG) and ventrolateral (vlPAG) subregions have been differentiated (Faull et al., 2019; Faull & Pattinson, 2017).

**TABLE 1:** Regions of interest.

Region of interest	Abbreviation	Origin
Dorsal anterior cingulate cortex	dACC	Harvard‐Oxford atlas, ‘anterior cingulate gyrus’, threshold = 30
Ventral anterior cingulate cortex	vACC	Harvard‐Oxford atlas, ‘subcallosal cortex’, threshold = 30
Anterior insula	aIC	Defined by (Wiech et al., 2014) based on published atlases and manual drawings
Posterior insula	pIC	Defined by (Wiech et al., 2014) based on published atlases and manual drawings
Dorsomedial prefrontal cortex	dmPFC	Harvard‐Oxford atlas, ‘superior frontal gyrus’, threshold = 30
Ventromedial prefrontal cortex	vmPFC	Harvard‐Oxford atlas, ‘frontal medial cortex’, threshold = 30
Amygdala	Amy	Harvard‐Oxford atlas, ‘amygdala’, threshold = 30
Primary motor cortex	M1	Harvard‐Oxford atlas, ‘precentral gyrus’, threshold = 30
Primary sensory cortex	S1	Harvard‐Oxford atlas, ‘postcentral gyrus’, threshold = 30
Lateral periaqueductal grey	lPAG	Functional seed location, MNI: −3, −32, −8 (Faull & Pattinson, 2017) with 2 mm sphere around it
Ventrolateral periaqueductal grey	vlPAG	Functional seed location, MNI: 7, −29, −8 (Faull & Pattinson, 2017) with 2 mm sphere around it

*Note*: Regions of interest (first row), regions of interest abbreviations (second row) and further information on how regions were created (Origins, third row). Threshold values relate to voxels with a probability of greater than 30% of being in a given structure.

The 4D time series were extracted within each region of interest mask by averaging over voxels. Functional connectivity measures were obtained by performing partial correlations between the 11 region of interest specific time series, to generate 55 imaging‐derived phenotypes (IDPs), which correspond to one half of the correlation matrix resulting from 11 regions of interest. IDPs were then winsorised at 1st and 99th percentiles to smooth over outliers, standardised and deconfounded by regressing effects of age, sex, imaging centre, head motion, head size, and table position out of the winsorised, normalised IDPs. By winsorising the data, extreme values do not have to be removed from the data set, but instead are trimmed. In this case, 1% of the extreme values (positive and negative) were set to the values of the 1st and 99th percentile (Gudivada et al., 2017). Standardisation of the data meant that for each variable, the mean was 0 and the standard deviation was 1.

### 2.3 Nonimaging derived phenotypes

Nonimaging derived phenotypes (nIDPs) were a set of 170 UK Biobank measures (see Table S1 for more details, and Table S2 for excluded variables) that broadly covered physiological/physical health (including also breathing, body measures, and bloods), mental health, and well‐being (also including measures related to anxiety, depression, and cognition) and lifestyle (including physical activity, smoking, nutrition, and job). Data were winsorised at 1st and 99th percentiles. Missing data were imputed (no variables had more than 50% of missing data, median: 1.98%, interquartile range: 3.62%, Figure S1 depicts a histogram of percentage missing data) using k‐nearest (k = 1) neighbour imputation. nIDPs were normalised and deconfounded (same as IDPs).

### 2.4 Planned statistical analyses

First, principal component analysis (PCA) was performed separately on IDPs (55 brain connectivity variables) and nIDPs (170 variables), identifying the top components that cumulatively explained 80% of the variance across all IDPs and nIDPs, respectively. These principal components were submitted to canonical correlation analysis (CCA) using MATLAB's *canoncorr* function. CCA is a multivariate technique to detect modes of covariation between two sets of variables. In this case the two sets were the PCA components derived from the IDPs and nIDPs (Miller et al., 2016; Smith et al., 2016). Modes represent linear combinations of the components in each set that maximally covary across subjects, termed *canonical variates*. CCA computed as many modes as there are components in the smaller set. To identify statistically significant modes (alpha level <.05 with family‐wise correction for multiple comparisons), a null distribution was established with 100,000 permutations of the subject rows of one set of components relative to the other. The strongest correlation from each permutation was selected to establish the null distribution. In order to interpret the modes with regard to the original variables, we took a conservative approach by creating averages of the two canonical variates and correlating these averaged variates with the (non‐PCA‐reduced) values for the IDPs and nIDPs. To facilitate interpretation, only correlation coefficients ≥0.2 and ≤ −0.2 were then reported (Smith et al., 2016). In addition, in order to assess how IDPs loaded onto individual modes, correlations of IDPs with canonical variates (see Figure S2) were multiplied by the sign of related population mean between node‐pair connectivity (see Figure S3 for related population mean between node‐pair connectivity).

## 3 RESULTS

**FIGURE 1:**
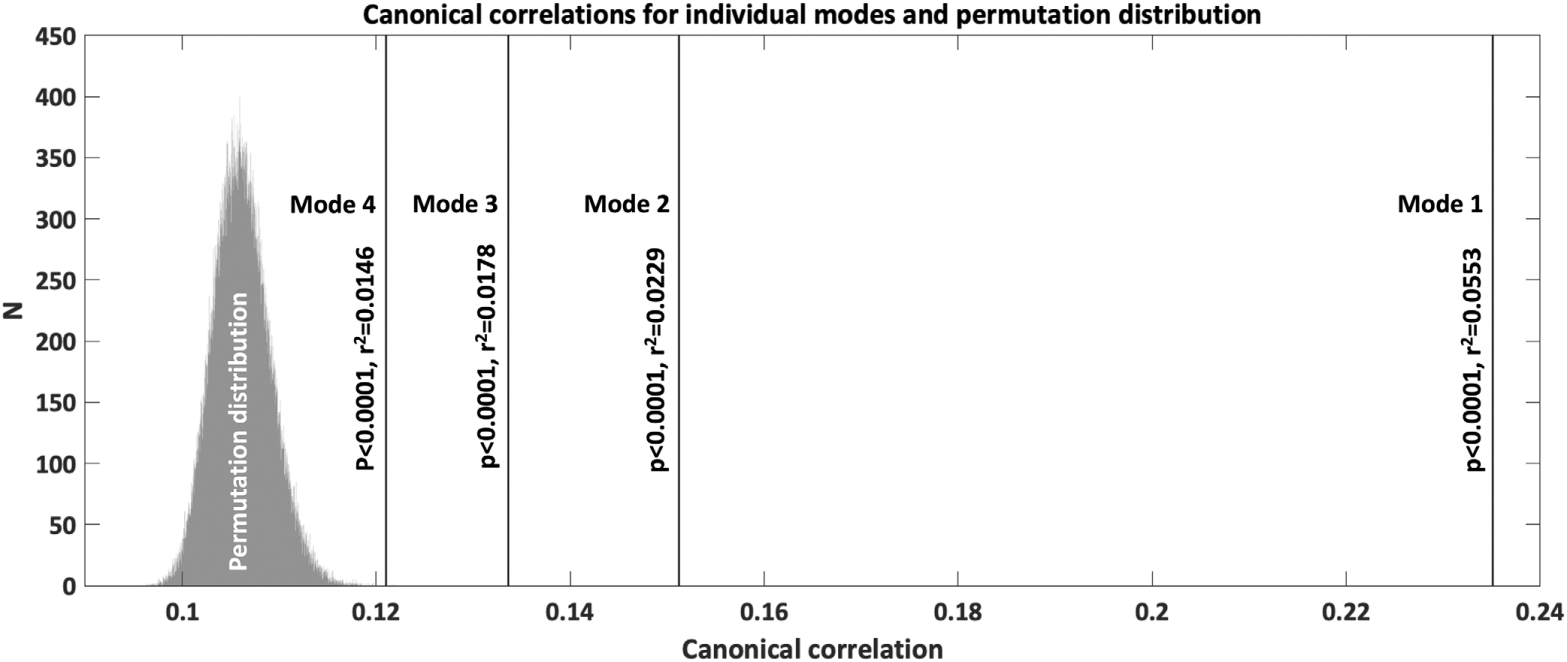
Canonical correlations for the four significant modes of co‐variation and permutation distribution. Canonical correlations for individual modes were evaluated against a null distribution of canonical correlations (grey bars) derived from permuting subject rows of one variable set relative to the other variable set 100,000 times. The strongest correlation from each permutation was selected to establish the permutation distribution. Vertical lines mark canonical correlations for the four modes of the original data which were significantly different from the null distribution (statistical significance and variance explained are shown).

After PCA, the top 33 components related to all IDPs cumulatively explained 81.38% of the variance. The top 88 components related to all nIDPs explained 80.37% of the variance. Those scores were used to calculate the canonical variates and resulting modes of covariation. Four modes of covariation were found to be statistically significant after permutation testing (Mode 1 canonical correlation: 0.2351, *p* < .0001, *r* ^2^ = .0553; Mode 2 canonical correlation: 0.1512, *p* < .0001, *r* ^2^ = .0229; Mode 3 canonical correlation: 0.1336, *p* < .0001, *r* ^2^ = .0178; Mode 4 canonical correlation: 0.1210, *p* < .0001, *r* ^2^ = .0146; Figure 1).

**FIGURE 2:**
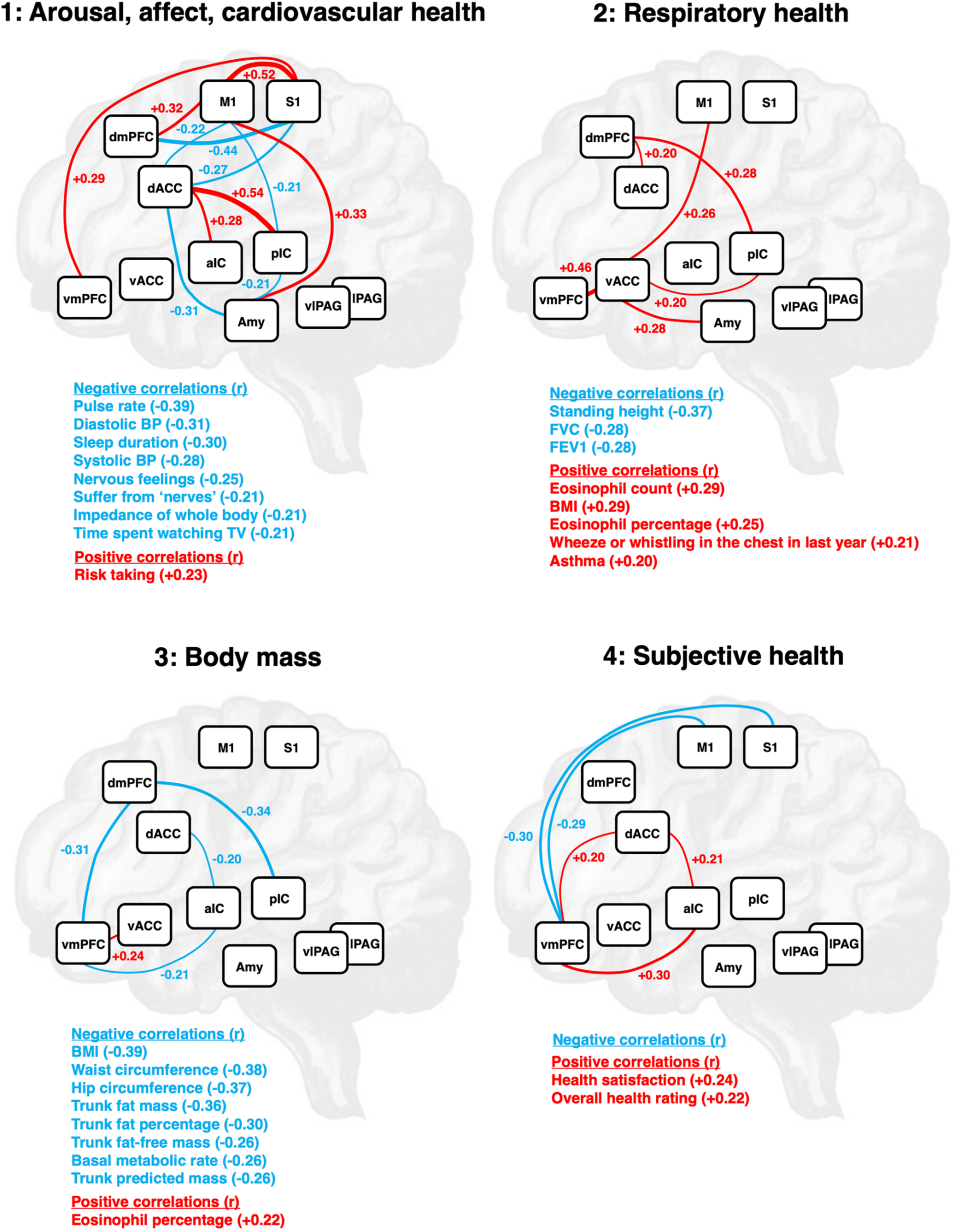
Significant modes: Neural circuits and associated nonimaging related phenotypes. Mode 1: Arousal, affect, cardiovascular health; *p* < .0001, variance explained: 5.53%. Mode 2: Respiratory health; *p* < .0001, variance explained: 2.29%. Mode 3: Body mass; *p* < .0001, variance explained: 1.78%. Mode 4: Subjective health; *p* < .0001, variance explained: 1.46%. For nonimaging related variables, negative correlations with canonical variates are depicted in blue, positive correlations in red. For better interpretation, correlations of imaging variables with canonical variates were multiplied by the sign of related population mean (node‐pair) correlations, so that red colour indicates stronger and blue colour weaker connections for high‐scoring subjects. Amy, amygdala; aIC, anterior insular cortex; BMI, body mass index; BP, blood pressure; dACC, dorsal anterior cingulate cortex; dmPFC, dorsomedial prefrontal cortex; FEV1, forced expiratory volume in 1 s; FVC, forced vital capacity; lPAG, lateral periaqueductal grey; M1, primary motor cortex; pIC, posterior insular cortex; S1, primary sensory cortex; vACC, ventral anterior cingulate cortex; vlPAG, ventrolateral periaqueductal grey; vmPFC, ventromedial prefrontal cortex.

CCA modes were correlated back into original individual variables and explained variance in subsets of original variables. We labelled Mode 1 (Figure 2: Mode 1) explaining 5.53% of the variance as related to arousal, affect and cardiovascular health, since nIDPs may primarily be associated with maladaptive arousal, affective processing and cardiovascular variables. The neural network of Mode 1 was very dense and characterised by prominent involvement of amygdala (3 connections), IC (4 connections), dACC (5 connections) and somatosensory and motor cortices (S1/M1, 8 connections).

Mode 2 (Figure 2: Mode 2) explaining 2.29% of the variance was labelled as related to respiratory health, since the set of nIDPs may predominantly be linked to breathing and respiratory disease. The neural network of mode 2 was centred at vACC (4 connections) with a comparably strong vACC‐vmPFC connection. pIC and dmPFC had each 2 connections.

We labelled Mode 3 (Figure 2: Mode 3) explaining 1.78% of the variance as being associated mainly with body mass (and metabolism). The neural network underlying Mode 3 included all prefrontal, insular, and anterior cingulate cortices. dmPFC featured 2 and vmPFC 3 connections.

Mode 4 (Figure 2: Mode 4) which explained 1.46% of the variance contained nIDPs of subjective health and was labelled accordingly. The neural network of Mode 4 contained predominantly vmPFC connections to somatosensory, motor, dorsal anterior cingulate, and anterior insular cortices. dACC featured 2 connections.

## 4 DISCUSSION

In 19,020 participants we identified four statistically independent modes of co‐variation between connectivity profiles within interoceptive brain regions and measures of lifestyle and health‐related functioning. Modes broadly represented distinct functional domains: Mode 1 related to arousal, affect and cardiovascular health, Mode 2 to respiratory health, Mode 3 to body mass and Mode 4 to subjective health. Modes go far beyond simple region‐function associations and represent specific ‘neural fingerprints’ of individual connectivity profiles associated with functional domains in the context of interoception. Our research contributes to the conceptualisation of interoception and sets the scope for future more directed and experimental research on the neural correlates of interoceptive involvement in various lifestyle and health‐related variables.

Our findings are conducive to a neurally informed characterisation of co‐morbid conditions. In one single analysis, we could relate functional connectivity to different health‐related domains outlining common and different mechanisms. For instance, Mode 1 shows a brain network linking variables of arousal, negative affect and cardiovascular health, supporting previous research suggesting a link between these dimensions at the neural level (Kraynak et al., 2018; Pollatos et al., 2007).

Furthermore, breathing pathology has been related to obesity (Hayen et al., 2013; Peters et al., 2018), negative affect and cardiovascular pathology (Harrison, Nanz, et al., 2021; Hayen et al., 2013; Richardson et al., 2006). In the present research, variables of respiratory health, body mass, and negative affect and cardiovascular health were to a large extent dissociated into mutually uncorrelated modes and different phenotypes of pathology may differ in the involvement of individual mode dimensions. However, Mode 2 related to respiratory health still contained variables arguably associated with body mass (BMI) and potentially affective processing (vACC‐Amygdala).

Arousal, affective processing related to anxiety, cardiovascular variables (Critchley & Garfinkel, 2017; Paulus & Stein, 2010; Pollatos et al., 2007) (Mode 1), breathing and breathing pathology (Harrison, Nanz, et al., 2021b; Marlow et al., 2019) (Mode 2), body weight and metabolic function (Quigley et al., 2021; Robinson et al., 2021) (Mode 3), and subjective health (Mode 4) and well‐being (Bonaz et al., 2021; Farb et al., 2015; Kananen et al., 2021) have previously been linked to interoception, and we suggest that further studies should consider their relation to interoception with regard to the different neural circuits highlighted by the present research.

As circuits share contributions of critical interoceptive and domain general insular, anterior cingulate, and medial prefrontal regions, pathological processing in these regions may have an impact on more than one functional domain. This could better explain why pathologies occur as co‐morbidities, and why interoception is relevant for a large variety of disorders (Bonaz et al., 2021; Kleckner et al., 2017). Our study could thus give rise to future research on a neurally informed classification of co‐morbid conditions and subjective health in the context of interoception cutting across traditional diagnostic boundaries (Cuthbert & Insel, 2013).

Neural circuits of individual modes were fully dissociated between Modes 1 and 2, and were generally specific representing distinct neural fingerprints of functional domains. This contributes to research on the conceptualisation of interoception, which describes and seeks to clarify relationships between different interoceptive functions associated with lower‐level sensing and regulation of bodily states and higher‐level emotional, cognitive, or even self‐related processing (Critchley & Garfinkel, 2017; Quigley et al., 2021). In the following, we will consider each mode individually.

### 4.1 Mode 1: Arousal, affect and cardiovascular health

Mode 1 may primarily be associated with arousal, affect and cardiovascular health. The majority of nIDPs related to cardiovascular health (‘Pulse rate’, ‘Diastolic BP’, ‘Systolic BP’) and negative affect, that is, anxiety (‘Nervous feelings’, ‘Suffer from nerves’) and correlated negatively with Mode 1. We suggest that these variables also signify a state of maladaptive arousal.

Autonomic arousal, as indicated by increased heart rate and blood pressure has been associated with anxiety related states (Kemp et al., 2014; Paterniti et al., 1999; Stevelink et al., 2020), although a negative association of systolic blood pressure with anxiety and depression has also been shown (Hildrum et al., 2008). It is theorised that bodily signals are crucial for affective experience (Craig, 2002; Critchley & Garfinkel, 2017; Damasio et al., 2000; James, 1994; Lange & Haupt, 1922; Seth & Friston, 2016), and high arousal in combination with negative valence may be a basis for symptoms of anxiety (Kuppens et al., 2013; Posner et al., 2005). ‘Sleep duration’ (Van Mill et al., 2010) and ‘Time spent watching TV’ (de Wit et al., 2011) have also previously been associated with anxiety. ‘Risk taking’ showed an inverse relationship with all other nIDPs, which seems reasonable given that calmer, less aroused, and distressed (Mano, 1992), less anxious (A. R. Smith, Ebert, & Broman‐Fulks, 2016a) subjects take more risky decisions.

We found a network including dACC, dmPFC, IC, sensorimotor and amygdala connections which were much more extensive than in any other mode. This seems plausible, as ACC, mPFC, IC and amygdala are involved in both autonomic and affective arousal (Satpute et al., 2019). In addition, dACC, dmPFC, IC and sensorimotor cortices are specifically important for the generation of cardiac interoceptive awareness (Critchley et al., 2004; Khalsa et al., 2009; Pollatos et al., 2007), can in that context partly relate to negative affect, including anxiety (Critchley et al., 2004; Pollatos et al., 2007), and also mediate cardiovascular arousal (Pollatos et al., 2007).

Amygdala connections were mainly found in this mode. Amygdala is critical for arousal and emotional processing, especially the processing of fear (Öhman, 2005; Phelps & LeDoux, 2005). Anxiety‐related states have been associated with a network containing amygdala, IC and/or ACC (Etkin & Wager, 2007; Gehrlach et al., 2019; Gold et al., 2015; Sehlmeyer et al., 2009), which were observed in the present research and may also form a circuit of negative affect relevant to threat dysregulation (Williams, 2016). In that respect, our findings are well in line with other research linking both negative emotions and variables of cardiovascular health to mPFC, ACC, IC, and amygdala (see Kraynak et al., 2018 for a review). We provide explicit connections between these regions at the population level that should inform future investigations examining the visceromotor and viscerosensory mechanisms linking affective states and physiological changes.

### 4.2 Mode 2: Respiratory health

We labelled Mode 2 as related to respiratory health. Major clinical respiratory variables ‘FVC’ (forced vital capacity) and ‘FEV1’ (forced expiratory volume in 1 s) (M. R. Miller et al., 2005) were negatively associated with canonical variates. ‘Standing height’ showed the same relationship, which is plausible given that taller subjects can exhale larger volumes. Variables related to respiratory disease included ‘Wheeze or whistling in the chest in the last year’, ‘Asthma’, ‘Eosinophil count’, ‘Eosinophil percentage’ and ‘BMI’ (body mass index) and showed a meaningful positive correlation with canonical variates. Eosinophils promote inflammation and are increased in some types of asthma (Wenzel, 2012). Obesity has been shown to be a major risk factor and disease modifier in asthma, where obese asthmatics show more frequent and severe exacerbations (Peters et al., 2018).

vACC connections were particularly prominent in Mode 2. A vACC‐vmPFC connection was especially strong and linked to variables of breathing pathology. This supports previous research in the context of chronic obstructive pulmonary disease, where vmPFC and vACC were associated with the evaluation of breathlessness and suggested to contribute to a poor correlation between lung function and symptoms (Herigstad et al., 2015). vACC‐amygdala connectivity was also linked to breathing pathology in Mode 2 and may be a target for future research, as amygdala processing has been associated with breathing inhibition (Rhone et al., 2020), and functional connections including amygdala and vACC are implicated in pathological emotional processing (Bijsterbosch et al., 2018; Connolly et al., 2013).

### 4.3 Mode 3: Body mass

Mode 3 was mainly related to body mass (and metabolism). ‘BMI’, ‘Waist circumference’, ‘Hip circumference’, ‘Trunk fat mass’, ‘Trunk fat percentage’, and ‘Basal metabolic rate’ were all part of Mode 3, correlating negatively with canonical variates. ‘Trunk predicted mass’ and ‘Trunk fat‐free mass’ correlated also negatively with canonical variates, where an association of body fat and fat‐free mass has been reported previously (Gray & Bauer, 1991). Eosinophils (‘Eosinophil percentage’) which are implicated in tissue homeostasis (Wu et al., 2011) correlated positively with canonical variates, but their relationship with body mass and obesity is under debate (Calco et al., 2020; Sunadome et al., 2020).

Physiological functioning requires the sensing, interpretation, and control of energy‐status‐related internal states, integrating them with energy needs, learned experiences, and exteroceptive information which motivates behavioural responses such as feeding behaviour (Berntson & Khalsa, 2021; Quigley et al., 2021). Therefore, it seems plausible that Mode 3 was characterised by a network including pIC, the major substrate of viscerosensation (Allen, 2020; Craig, 2009; Kurth et al., 2010), vACC and aIC, the primary regions of visceromotor control (Craig, 2009; Kleckner et al., 2017), and dorsal and ventral medial prefrontal cortices controlling affective and cognitive processes and behaviour (Kensinger & Ford, 2021; Roy et al., 2012; Venkatraman & Huettel, 2012).

Functional connectivity modulations in brain structures including vmPFC, dmPFC, dACC, and aIC were shown in obese patients following bariatric surgery (Li et al., 2018), and our research suggests that a network including these regions may play a more general role in body mass. However, contrary to previous research investigating resting‐state connectivity in obese participants (Donofry et al., 2020), dmPFC‐pIC connectivity, exhibiting the strongest modulation with Mode 3, was shown to increase with BMI in our population level sample of participants.

### 4.4 Mode 4: Subjective health

Mode 4 was related to variables of subjective health including ‘Health satisfaction’ and ‘Overall health rating’. Variables of subjective health should involve processes related to self‐reference, appraisal and emotion. Crucially, vmPFC was the centre of the network of Mode 4 and connected to every other region of the network. This seems plausible, as many different functions that have been associated with vmPFC have been summarised to relate to subjective value estimation (Levy & Glimcher, 2012), specifically in relation to the self (D'Argembeau, 2013), and the contextual shaping of affective information (Roy et al., 2012). Subjective health is an important indicator to describe the actual needs and problems of patients and can reflect and influence physiology. Interestingly, vmPFC plays a central role in mediating the interplay between self‐related, also interoceptive states and physiology (Koban et al., 2021), and we provide explicit vmPFC connections for future more directed research.

### 4.5 Strengths and limitations

The major strength of the study is the sample size of more than 19,000 participants (Littlejohns et al., 2020) which has allowed us to obtain meaningful and robust hidden associations between two sets of variables using CCA (Hotelling, 1992; Wang et al., 2020). Our data‐driven approach enabled a truly exploratory analysis within the boundaries of pre‐selected interoceptive regions, linking specific brain networks with a possible relation to interoception to a broad range of specific correlated lifestyle and health‐related variables. Although our sample size facilitates the detection of small effects, the four modes identified together represent more than 10% of population variance. The identification of multiple significant modes of population variance was not a foregone conclusion. For example, Smith et al. (2015) who used CCA to analyse multivariable whole brain connectivity and diverse health‐relevant nonbrain data in 461 subjects from the Human Connectome Project obtained only one strong mode.

There is a large correspondence between resting‐state and task‐related networks (Smith et al., 2009), and resting state activity can shape task activations (Cole et al., 2016) and predict behaviour (Zou et al., 2013). As our results were not constrained by a specific task, we could employ one integrative analysis relating functional connectivity to different health‐related fields and delineating shared and separated mechanisms. Consequently, our research contributes to a biologically informed, brain‐based characterisation of co‐morbid conditions. At the same time, future research aiming to investigate interoceptive involvement in specific individual domains may also study task‐induced functional connectivity, which might still better relate to specific nonimaging traits (Greene et al., 2018; Jiang et al., 2020). For instance, the functional connectome of regions relevant for interoception during heartbeat (Kleckner et al., 2015) or filter (Harrison, Garfinkel, et al., 2021) detection tasks, could still further elucidate interoceptive involvement in cardiovascular or respiratory health, respectively.

The present large‐scale data set is observational and cross‐sectional, so that causal interpretations of our results are not possible. In addition, the data set does not contain explicit measures of interoception. As brain regions relevant to interoception are also relevant to other functions, we cannot draw definite conclusions about interoception (Poldrack, 2006). However, we relate specific connectivity profiles within interoceptive brain regions to specific health‐related domains. These domain‐specific neural fingerprints can contribute to conceptualizing interoception (Critchley & Garfinkel, 2017; Quigley et al., 2021) and inform future experimental interoceptive research.

We included lPAG and vlPAG as previous research suggests that the PAG is a brain structure involved in interoception, information integration, and autonomic behavioural control, crucially in the context of breathing (Faull et al., 2019; Faull & Pattinson, 2017). However, we did not show resting PAG connectivity with breathing Mode 2 or any other mode. As lPAG and vlPAG ROIs were necessarily very small, a low signal‐to‐noise ratio may have prevented the detection of an effect.

Our results are inevitably constrained by the selection of variables. We pre‐selected a set of areas identified in previous studies relevant to interoception. Nonimaging phenotypes were selected very broadly to chart neural variables of various lifestyle and health‐related conditions. Due to the breadth of variables and novelty of our approach variable selection was necessarily subject to educated judgement. Upcoming investigations should therefore aim to replicate and extend our findings employing variations in the selection of imaging and nonimaging related variables.

## 5 CONCLUSION

To conclude, we studied the relationship of a functional network of brain regions relevant to interoception to a broad selection of nonimaging health and lifestyle‐related phenotypes in more than 19,000 UK Biobank study participants. Our integrative and data driven analysis revealed four modes of population co‐variation with distinct neural circuits relating to interoception and respectively arousal and affect and cardiovascular health, respiratory health, body mass or subjective health. Circuits can be regarded as specific neural ‘fingerprints’ of functional domains and set the scope for more directed research on the neurobiology of interoceptive involvement in different lifestyle and health‐related factors. Therefore, our research contributes to the conceptualisation of interoception and may help to better understand co‐morbid conditions in the light of shared interoceptive structures.

## FUNDING INFORMATION

Micah Allen was supported by a Lundbeckfonden Fellowship (under Grant [R272‐2017‐4345]), and a grant from the European Research Council (ERC‐StG‐948788).

## CONFLICT OF INTEREST STATEMENT

The authors declare no competing financial interests.

## Supporting information


**Data S1:** Supporting Information

## Data Availability

Access to the raw data of the present research can be provided by UK Biobank upon successful application (Sudlow et al., 2015).
