# Supplementary material for: Functional connectivity between interoceptive brain regions is associated with distinct health‐related domains: A population‐based neuroimaging study
Source: Hum Brain Mapp. 2023 Mar 20;44(8):3210–21. doi: 10.1002/hbm.26275 (PMC10171512; doi:10.1002/hbm.26275)
Supplement: Supplementary file 1 — Data S1: Supporting Information [file HBM-44-3210-s001.docx]

**Supplemental information**

**Table S1. Full list of 170 selected nIDPs**

| **Category** | **Variable name** | **Biobank code** |
| --- | --- | --- |
|  |  |  |
| **Physiological health** |  |  |
| *Various* | Pulse rate, automated reading | 102 |
|  | Number of self-reported cancers | 134 |
|  | Number of self-reported non-cancer illnesses | 135 |
|  | Number of operations, self-reported | 136 |
|  | Long-standing illness, disability or infirmity | 2188 |
|  | Chest pain or discomfort | 2335 |
|  | Diabetes diagnosed by doctor | 2443 |
|  | Cancer diagnosed by doctor | 2453 |
|  | Fractured/broken bones in last 5 years | 2463 |
|  | Other serious medical condition/disability diagnosed by doctor | 2473 |
|  | Diastolic blood pressure, automated reading | 4079 |
|  | Systolic blood pressure, automated reading | 4080 |
| *Breathing* | Wheeze or whistling in the chest in last year | 2316 |
|  | Peak expiratory flow (PEF) | 3064 |
|  | Shortness of breath walking on level ground | 4717 |
|  | asthma | 6152 |
|  | blood clot in leg | 6152 |
|  | blood clot in lung | 6152 |
|  | Hayfever, allergic rhinitis or eczema | 6152 |
|  | Forced expiratory volume in 1-second (FEV1), Best measure | 20150 |
|  | Forced vital capacity (FVC), Best measure | 20151 |
|  | Cough on most days | 22502 |
|  | Years of cough on most days | 22503 |
|  | Bring up phlegm/sputum/mucus on most days | 22504 |
|  | Years of bringing up phlegm/sputum/mucus on most days | 22505 |
|  | FEV1/FVC ratio | calculated |
| *Body measures* | Waist circumference | 48 |
|  | Hip circumference | 49 |
|  | Standing height | 50 |
|  | Body mass index (BMI) | 21001 |
|  | Basal metabolic rate | 23105 |
|  | Impedance of whole body | 23106 |
|  | Trunk fat percentage | 23127 |
|  | Trunk fat mass | 23128 |
|  | Trunk fat-free mass | 23129 |
|  | Trunk predicted mass | 23130 |
| *Bloods* | White blood cell (leukocyte) count | 30000 |
|  | Red blood cell (erythrocyte) count | 30010 |
|  | Haemoglobin concentration | 30020 |
|  | Haematocrit percentage | 30030 |
|  | Mean corpuscular volume | 30040 |
|  | Mean corpuscular haemoglobin | 30050 |
|  | Mean corpuscular haemoglobin concentration | 30060 |
|  | Red blood cell (erythrocyte) distribution width | 30070 |
|  | Platelet count | 30080 |
|  | Platelet crit | 30090 |
|  | Mean platelet (thrombocyte) volume | 30100 |
|  | Platelet distribution width | 30110 |
|  | Lymphocyte count | 30120 |
|  | Monocyte count | 30130 |
|  | Neutrophill count | 30140 |
|  | Eosinophill count | 30150 |
|  | Basophill count | 30160 |
|  | Nucleated red blood cell count | 30170 |
|  | Lymphocyte percentage | 30180 |
|  | Monocyte percentage | 30190 |
|  | Neutrophill percentage | 30200 |
|  | Eosinophill percentage | 30210 |
|  | Basophill percentage | 30220 |
|  | Nucleated red blood cell percentage | 30230 |
|  | Reticulocyte percentage | 30240 |
|  | Reticulocyte count | 30250 |
|  | Mean reticulocyte volume | 30260 |
|  | High light scatter reticulocyte percentage | 30290 |
|  | High light scatter reticulocyte count | 30300 |
| **Mental health and well-being** |  |  |
| *Various* | Mood swings | 1920 |
|  | Miserableness | 1930 |
|  | Irritability | 1940 |
|  | Sensitivity / hurt feelings | 1950 |
|  | Fed-up feelings | 1960 |
|  | Nervous feelings | 1970 |
|  | Worrier / anxious feelings | 1980 |
|  | Tense / ''highly strung'' | 1990 |
|  | Worry too long after embarrassment | 2000 |
|  | Suffer from ''nerves'' | 2010 |
|  | Loneliness, isolation | 2020 |
|  | Guilty feelings | 2030 |
|  | Risk taking | 2040 |
|  | Frequency of depressed mood in last 2 weeks | 2050 |
|  | Frequency of unenthusiasm / disinterest in last 2 weeks | 2060 |
|  | Frequency of tenseness / restlessness in last 2 weeks | 2070 |
|  | Frequency of tiredness / lethargy in last 2 weeks | 2080 |
|  | Seen doctor (GP) for nerves, anxiety, tension or depression | 2090 |
|  | Seen a psychiatrist for nerves, anxiety, tension or depression | 2100 |
|  | Able to confide | 2110 |
|  | Overall health rating | 2178 |
|  | Happiness | 4526 |
|  | Work/job satisfaction | 4537 |
|  | Health satisfaction | 4548 |
|  | Family relationship satisfaction | 4559 |
|  | Friendships satisfaction | 4570 |
|  | Financial situation satisfaction | 4581 |
|  | Ever depressed for a whole week | 4598 |
|  | Longest period of depression | 4609 |
|  | Number of depression episodes | 4620 |
|  | Ever unenthusiastic/disinterested for a whole week | 4631 |
|  | Ever manic/hyper for 2 days | 4642 |
|  | Ever highly irritable/argumentative for 2 days | 4653 |
|  | Neuroticism score | 20127 |
| *Cognition* | Number of correct matches in round | 398 |
|  | Number of incorrect matches in round | 399 |
|  | Time to complete round | 400 |
|  | Time to answer | 4288 |
|  | Number of attempts | 4291 |
|  | Fluid intelligence score | 20016 |
|  | Prospective memory result | 20018 |
|  | Mean time to correctly identify matches | 20023 |
|  | Fluid intelligence questions attempted within time limit | 20128 |
| **Lifestyle** |  |  |
| *Physical activity* | Number of days/week walked 10+ minutes | 864 |
|  | Duration of walks | 874 |
|  | Number of days/week of moderate physical activity 10+ minutes | 884 |
|  | Duration of moderate activity | 894 |
|  | Number of days/week of vigorous physical activity 10+ minutes | 904 |
|  | Duration of vigorous activity | 914 |
|  | Usual walking pace | 924 |
|  | Frequency of stair climbing in last 4 weeks | 943 |
|  | Frequency of walking for pleasure in last 4 weeks | 971 |
|  | Duration walking for pleasure | 981 |
|  | Frequency of light DIY in last 4 weeks | 1011 |
|  | Duration of light DIY | 1021 |
|  | Time spent outdoors in summer | 1050 |
|  | Time spent outdoors in winter | 1060 |
|  | Time spent watching television | 1070 |
|  | Time spent using computer | 1080 |
|  | Time spent driving | 1090 |
|  | Drive faster than motorway speed limit | 1100 |
|  | Sleep duration | 1160 |
|  | Frequency of heavy DIY in last 4 weeks | 2624 |
|  | Duration of heavy DIY | 2634 |
|  | Frequency of other exercises in last 4 weeks | 3637 |
|  | Duration of other exercises | 3647 |
| *Smoking* | Current tobacco smoking | 1239 |
|  | Past tobacco smoking | 1249 |
|  | Smoking/smokers in household | 1259 |
|  | Exposure to tobacco smoke at home | 1269 |
|  | Exposure to tobacco smoke outside home | 1279 |
|  | Smoking status | 20116 |
|  | Ever smoked | 20160 |
| *Nutrition* | Cooked vegetable intake | 1289 |
|  | Salad / raw vegetable intake | 1299 |
|  | Fresh fruit intake | 1309 |
|  | Dried fruit intake | 1319 |
|  | Oily fish intake | 1329 |
|  | Non-oily fish intake | 1339 |
|  | Processed meat intake | 1349 |
|  | Poultry intake | 1359 |
|  | Beef intake | 1369 |
|  | Lamb/mutton intake | 1379 |
|  | Pork intake | 1389 |
|  | Cheese intake | 1408 |
|  | Bread intake | 1438 |
|  | Cereal intake | 1458 |
|  | Tea intake | 1488 |
|  | Coffee intake | 1498 |
|  | Water intake | 1528 |
|  | Alcohol intake frequency | 1558 |
|  | Average weekly red wine intake | 1568 |
|  | Average weekly champagne plus white wine intake | 1578 |
|  | Average weekly beer plus cider intake | 1588 |
|  | Average weekly spirits intake | 1598 |
|  | Average weekly fortified wine intake | 1608 |
|  | Alcohol usually taken with meals | 1618 |
|  | Alcohol drinker status | 20117 |
| *Job* | Townsend deprivation index at recruitment | 189 |
|  | Time employed in main current job | 757 |
|  | Length of working week for main job | 767 |
|  | Job involves mainly walking or standing | 806 |
|  | Job involves heavy manual or physical work | 816 |
|  | Job involves shift work | 826 |
|  | Age completed full time education | 845 |
|  |  |  |
|  |  |  |

If variables featured more than one data point the most recent data point was selected or, if appropriate, an average measure was calculated. In some cases, variables were re-coded for consistency and to fit the analysis structure. Unclear information from categories of individual variables (e.g. “Prefer not to say”, “Do not know”) was excluded from the analysis.

**Table S2. Full list of 107 excluded nIDPs**

| **Reason for exclusion** | **Variable name** | **Biobank code** |
| --- | --- | --- |
|  |  |  |
| **Used for unconfounding** | Year of birth | 34 |
|  | Month of birth | 52 |
|  | Scan date | 53 |
|  | Sex | 31 |
| **Duplicates** | Systolic blood pressure, manual reading | 93 |
|  | Diastolic blood pressure, manual reading | 94 |
|  | Pulse rate (during blood-pressure measurement) | 95 |
|  | Forced vital capacity (FVC) | 3062 |
|  | Forced expiratory volume in 1-second (FEV1) | 3063 |
|  | Average monthly red wine intake | 4407 |
|  | Average monthly champagne plus white wine intake | 4418 |
|  | Average monthly beer plus cider intake | 4429 |
|  | Average monthly spirits intake | 4440 |
|  | Average monthly fortified wine intake | 4451 |
|  | Average monthly intake of other alcoholic drinks | 4462 |
|  | Doctor diagnosed hayfever or allergic rhinitis | 22126 |
|  | Doctor diagnosed asthma | 22127 |
|  | Year of birth | 22200 |
|  | Tobacco smoking | 22506 |
|  | BMI | 23104 |
| **Postcode dependent variables *** | Frequency of travelling from home to job workplace | 777 |
|  | Distance between home and job workplace | 796 |
|  | Frequency of friend/family visits | 1031 |
|  | Nitrogen dioxide air pollution; 2010 | 24003 |
|  | Nitrogen oxides air pollution; 2010 | 24004 |
|  | Particulate matter air pollution (pm10); 2010 | 24005 |
|  | Particulate matter air pollution (pm2.5); 2010 | 24006 |
|  | Particulate matter air pollution (pm2.5) absorbance; 2010 | 24007 |
|  | Particulate matter air pollution 2.5-10um; 2010 | 24008 |
|  | Traffic intensity on the nearest road | 24009 |
|  | Inverse distance to the nearest road | 24010 |
|  | Traffic intensity on the nearest major road | 24011 |
|  | Inverse distance to the nearest major road | 24012 |
|  | Total traffic load on major roads | 24013 |
|  | Close to major road | 24014 |
|  | Sum of road length of major roads within 100m | 24015 |
|  | Nitrogen dioxide air pollution; 2005 | 24016 |
|  | Nitrogen dioxide air pollution; 2006 | 24017 |
|  | Nitrogen dioxide air pollution; 2007 | 24018 |
|  | Particulate matter air pollution (pm10); 2007 | 24019 |
| **Structural brain variables **** | Volumetric scaling from T1 head image to standard space | 25000 |
|  | Volume of peripheral cortical grey matter (normalised for head size) | 25001 |
|  | Volume of peripheral cortical grey matter | 25002 |
|  | Volume of ventricular cerebrospinal fluid (normalised for head size) | 25003 |
|  | Volume of ventricular cerebrospinal fluid | 25004 |
|  | Volume of grey matter (normalised for head size) | 25005 |
|  | Volume of grey matter | 25006 |
|  | Volume of white matter (normalised for head size) | 25007 |
|  | Volume of white matter | 25008 |
| **<1% confirmed events** | Used an inhaler for chest within last hour | 3090 |
|  | Average weekly intake of other alcoholic drinks | 5364 |
|  | Previously smoked cigarettes on most/all days | 5959 |
|  | Emphysema/chronic bronchitis | 6152 |
|  | Number of cigarettes previously smoked daily (current cigar/pipe smoker) | 6183 |
|  | Age stopped smoking cigarettes (current cigar/pipe or previous cigarette smoker) | 6194 |
|  | Doctor diagnosed emphysema | 22128 |
|  | Doctor diagnosed chronic bronchitis | 22129 |
|  | Doctor diagnosed COPD (chronic obstructive pulmonary disease) | 22130 |
|  | Doctor diagnosed cystic fibrosis | 22131 |
|  | Doctor diagnosed alpha-1 antitrypsin deficiency | 22132 |
|  | Doctor diagnosed sarcoidosis | 22133 |
|  | Doctor diagnosed bronchiectasis | 22134 |
|  | Doctor diagnosed idiopathic pulmonary fibrosis | 22135 |
|  | Doctor diagnosed fibrosing alveolitis/unspecified alveolitis | 22136 |
|  | Doctor diagnosed tuberculosis | 22137 |
|  | Doctor diagnosed silicosis | 22138 |
|  | Doctor diagnosed asbestosis | 22139 |
|  | Doctor diagnosed lung cancer (not mesothelioma) | 22140 |
|  | Doctor diagnosed mesothelioma of the lung | 22141 |
| **>50% missing data** | Frequency of strenuous sports in last 4 weeks | 991 |
|  | Duration of strenuous sports | 1001 |
|  | Light smokers, at least 100 smokes in lifetime | 2644 |
|  | Age started smoking in former smokers | 2867 |
|  | Number of cigarettes previously smoked daily | 2887 |
|  | Age stopped smoking | 2897 |
|  | Ever stopped smoking for 6+ months | 2907 |
|  | General pain for 3+ months | 2956 |
|  | Neck/shoulder pain for 3+ months' | 3404 |
|  | Hip pain for 3+ months | 3414 |
|  | Job involve night shift work | 3426 |
|  | Age started smoking in current smokers | 3436 |
|  | Number of cigarettes currently smoked daily (current cigarette smokers) | 3456 |
|  | Back pain for 3+ months | 3571 |
|  | Chest pain or discomfort walking normally | 3606 |
|  | Chest pain due to walking ceases when standing still | 3616 |
|  | Chest pain or discomfort when walking uphill or hurrying | 3751 |
|  | Knee pain for 3+ months | 3773 |
|  | Age asthma diagnosed | 3786 |
|  | Headaches for 3+ months | 3799 |
|  | Facial pains for 3+ months | 4067 |
|  | Maximum digits remembered correctly | 4282 |
|  | Longest period of unenthusiasm / disinterest | 5375 |
|  | Number of unenthusiastic/disinterested episodes | 5386 |
|  | Length of longest manic/irritable episode | 5663 |
|  | Severity of manic/irritable episodes | 5674 |
|  | Doctor restricts physical activity due to heart condition | 6014 |
|  | Chest pain felt during physical activity | 6015 |
|  | Chest pain felt outside physical activity | 6016 |
|  | Able to walk or cycle unaided for 10 minutes | 6017 |
|  | Maximum workload during fitness test | 6032 |
|  | Maximum heart rate during fitness test | 6033 |
|  | Single episode of probable major depression | 20123 |
|  | Probable recurrent major depression (moderate) | 20124 |
|  | Probable recurrent major depression (severe) | 20125 |
|  | Bipolar and major depression status | 20126 |
|  | Age of stopping smoking | 22507 |
|  | Amount of tobacco currently smoked | 22508 |

* Postcode dependent variables may not be representative as moving houses can strongly influence these variables.

** Structural brain variables were not the focus of the present investigation.

Variables used for unconfounding, duplicates, postcode dependent variables and structural brain variables were excluded before excluding variables according to rare events (<1%) and then missing datasets (>50%, see also Figure S1 below).

**Figure S1.** % missing datasets for non-brain variables before excluding variables according to missing data. Variables with more than 50% missing data were excluded.

**Figure S2.** Correlations of IDPs with canonical variates of individual Modes 1-4. Abbreviations: Amy (amygdala), aIC (anterior insular cortex), dACC (dorsal anterior cingulate cortex), dmPFC (dorsomedial prefrontal cortex), lPAG (lateral periaqueductal grey), M1 (primary motor cortex), pIC (posterior insular cortex), S1 (primary sensory cortex), vACC (ventral anterior cingulate cortex), vlPAG (ventrolateral periaqueductal grey), vmPFC (ventromedial prefrontal cortex). Negative correlations with canonical variates are depicted in blue, positive correlations in red.

**Figure S3.** Population mean raw functional connectivity (partial correlation) between node-pairs of IDPs correlating with any of the 4 modes. Abbreviations: Amy (amygdala), aIC (anterior insular cortex), dACC (dorsal anterior cingulate cortex), dmPFC (dorsomedial prefrontal cortex), M1 (primary motor cortex), pIC (posterior insular cortex), S1 (primary sensory cortex), vACC (ventral anterior cingulate cortex), vmPFC (ventromedial prefrontal cortex). Red colour indicates positive, blue colour negative functional connectivity between node-pairs.
